# Delivery characteristics of a low-resistance dry-powder inhaler used to deliver the long-acting muscarinic antagonist glycopyrronium*

**DOI:** 10.3109/21556660.2013.766197

**Published:** 2013-02-01

**Authors:** Paul Colthorpe, Thomas Voshaar, Thomas Kieckbusch, Erika Cuoghi, Juergen Jauernig

**Affiliations:** 1 Novartis Pharma AG, BaselSwitzerland; 2 Krankenhaus Bethanien, Moers, IMS Health FrankfurtGermany

**Keywords:** COPD, Dry-powder inhaler, Glycopyrronium, Tiotropium

## Abstract

**Objectives:**

The long-acting muscarinic antagonist (LAMA) glycopyrronium (NVA237) has recently been approved as a once-daily treatment for COPD. The objectives of this study were to determine the dose delivery characteristics of glycopyrronium and compare them with those of the LAMA tiotropium, both delivered by their respective capsule-based dry-powder inhalers (DPIs).

**Research design and methods:**

Seven inhalation profiles derived from patients with moderate and severe COPD were reproduced to determine the aerodynamic particle size distribution of glycopyrronium delivered by the Breezhaler device, a low-resistance DPI†. Theoretical respiratory tract deposition was estimated using a semi-empirical model for healthy lungs. These results were compared with those of tiotropium delivered by the high-resistance HandiHaler‡ device obtained in a previous study using the same set of inhalation profiles. Study limitations are that fine particle fraction (FPF) and particle size are generated by the inhalers are not a direct measure of lung deposition, and the bronchodilator effect of inhaled drugs does not depend solely upon the percentage of the total dose that reaches the lung.

**Results:**

The mean FPF (≤4.7 µm) was 42.6% of the nominal dose (which refers to the content of the capsule) for glycopyrronium and 9.8% for tiotropium while the mass median aerodynamic diameter (MMAD) was 2.8 µm and 3.9 µm for glycopyrronium and tiotropium, respectively. The mean estimated intrathoracic drug deposition as a percentage of the mean dose delivered to the Next Generation Impactor was 39% for glycopyrronium and 22% for tiotropium.

**Conclusions:**

The glycopyrronium capsule-based DPI delivered a higher FPF and greater and more consistent intrathoracic deposition irrespective of age and disease severity compared to the tiotropium capsule-based DPI, suggesting that it may be suitable for use by patients with a wide range of COPD severities.

## Introduction

Chronic obstructive pulmonary disease (COPD), a preventable lung disease characterized by progressive airflow limitation, is a leading cause of mortality and morbidity world-wide^1^. Bronchodilators are the mainstay of treatment of COPD, with inhalation preferred over other routes of administration because of greater efficacy and safety^2^. Pressurized metered-dose inhalers (pMDIs) and dry-powder inhalers (DPIs) are the main two types of inhaler currently available^3^. The Respimat inhaler, is a third type of device, which uses the mechanical energy of a spring to atomize the drug solution by forcing it through an extremely fine nozzle system, generating a slow-moving fine mist^5^. The device requires the patient to coordinate inhalation with actuation.

DPIs, in which the patient’s inspiratory flow provides the force to generate the drug aerosol, have become increasingly popular in COPD as the lack of the need to co-ordinate actuation and inhalation is important in minimizing patient handling errors and optimizing drug delivery^4^.

The internal resistance of the DPI determines the effort patients have to make to achieve adequate inspiratory flows for effective and reproducible dose delivery^6,7^. As high-resistance devices require greater effort to generate these flows, they may not be suitable for patients with significant airflow obstruction^8,9^.

In addition to providing consistent drug delivery over a wide range of inspiratory flow rates, an ideal inhaler for use in COPD should also generate optimal particle size for lung delivery and retention at the required site^10^. The aerodynamic size of drug particles generated by inhalers is one of the most important factors in defining the distribution and deposition of drug within the lung, with a particle size of less than 4.7 µm in diameter generally considered optimal for deposition in the bronchi and alveoli^11^. A high fine particle fraction (FPF), defined as fraction of particles of less than 4.7 µm in diameter, indicates that a significant proportion of the inhaled dose is likely to reach the pulmonary region.

Inhaled long-acting β_2_-agonists (LABAs) and long-acting muscarinic antagonists (LAMAs) are two classes of bronchodilator widely used for the treatment of patients with moderate-to-severe COPD^1,12,13^. Indacaterol maleate is a once-daily LABA delivered by the low-resistance DPI Breezhaler*^14^. This device has been shown to deliver a consistent dose of indacaterol, irrespective of disease severity and associated inhalation flow profiles^15^. The once-daily LAMA tiotropium is delivered by the high-resistance HandiHaler* device.

Glycopyrronium, a once-daily LAMA recently approved as a dry-powder formulation for the treatment of COPD, is also delivered via the same type of device currently used with indacaterol. To evaluate the dose delivery characteristics of glycopyrronium by this capsule-based DPI, the aerodynamic particle size distribution was determined and the theoretical respiratory tract deposition estimated under simulated inhalation conditions. Inspiratory profiles were obtained from seven patients representative of a wide range of COPD severities, ages, and degrees of airflow obstruction. The results for glycopyrronium obtained in this study were also compared with those for tiotropium delivered by its capsule-based DPI generated in a previous study using the same set of inhalation profiles^16^.

## Methods

**Figure 1.**
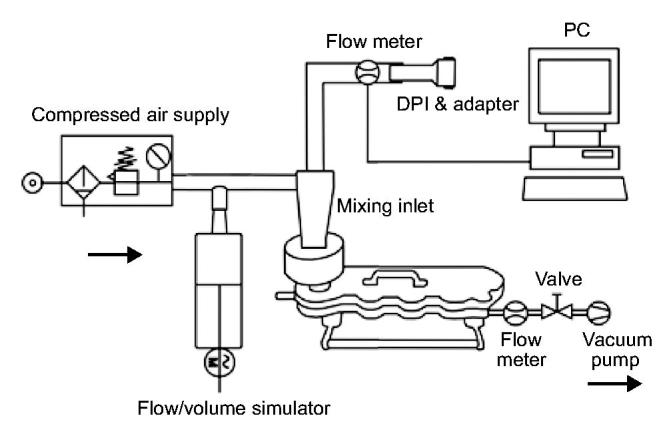
Experimental set-up with flow/volume simulator. DPI, dry-powder inhaler. Data for the tiotropium capsule-based DPI were obtained Chapman *et al*.^16^.

Seven patient inhalation profiles were selected in a previous study from a group of 26 patients to cover a range from moderate to severe COPD and represent different degrees of airflow obstruction^16^. The generation of the inhalation flow profiles was performed with an empty device for glycopyrronium and with a device containing the drug product for tiotropium. The profiles were reproduced in a flow/volume simulator (Hans Rudolph, Inc., USA) coupled through a mixing inlet to a Next Generation Impactor (NGI, Copley Scientific, UK) with pre-separator and induction port (Figure 1). Selected flow profiles were limited to a peak inspiratory flow of 100 L/min at maximum due to technical limitations of the experimental design (e.g., NGI calibration for a maximum flow rate at 100 L/min, limited maximum flow rate generated by flow/volume simulator). The flow/volume simulator was used to reproduce flow profiles by balancing and modulating the air flow through the DPI and auxiliary air to maintain a constant flow rate of 100 L/min through the NGI. The patient’s breathing patterns were reproduced at the mouthpiece of the DPIs by modulating the air flow using the computer-controlled flow volume simulator. At the impactor outlet, a flow rate of 100 L/min was generated by means of a vacuum pump whereas an air supply of 100 L/min leading into the mixing inlet assured a neutral sum flow at experimental rest as previously described by Chapman *et al.*^16^. Three replicate measurements were performed for each simulated patient flow profile, using a new DPI for each determination. Each capsule contained 50 µg of glycopyrronium (nominal dose).

To determine glycopyrronium deposition, the NGI was disassembled and samples from each component were analyzed by high-performance liquid chromatography. Data evaluation was carried out using validated software from Copley (C.I.T.D.A.S., Version 2.0). The delivered dose (DD) to the NGI was defined as drug amount from mouth piece adapter, USP throat, mixing inlet, pre-separator and all NGI collection cups. The fine particle mass (FPM) was calculated for an aerodynamic diameter of ≤4.7 μm to allow comparison with data obtained for tiotropium^16^. The fine particle fraction (FPF) was calculated by expressing the quotient of FPM relative to the nominal dose (contents of the capsule), which was 50 μg for glycopyrronium. The mass median aerodynamic diameter (MMAD) and geometric standard deviation (GSD), a measure of the variability of the particle diameters within the aerosol, were also determined.

Based on the results of the particle size analysis, theoretical respiratory tract depositions were estimated for each of the patient’s breathing profiles using the mathematical ICRP-lung model ICRP 66, which estimates extrathoracic and intrathoracic deposition in healthy subjects^17^. Extrathoracic deposition equals the deposition in the mouth cavity and larynx. Intrathoracic deposition equals the deposition in trachea, bronchial tree and alveolar region.

The original model was designed for continuous drug delivery during inhalation. It was adapted for inhalation with bolus drug delivery by simulating three different depositions using total inhaled volume (*D*_1_), volume until the beginning of the bolus (*D*_2_) and volume until the end of the bolus (*D*_3_). The deposition of the bolus (*D*_bolus_) was calculated using the following equation:
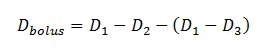


Input variables were MMAD, GSD, mean inhalation flow, inhalation volume, onset time of aerosol bolus, aerosol bolus length, functional residual capacity (FRC), age and breath hold after inhalation. As the model assumes a constant inhalation flow rate, the patients’ actual inhalation flow rate was converted to a constant mean inhalation flow rate over the entire flow rate values. Mean flow rate until the beginning of the bolus and bolus volume were assumed to be 12 L/min and 500 ml, respectively. These parameters were determined empirically by Chapman *et al*.^16^ but not confirmed *in vitro* for the two inhalers. The *in vitro* dose delivery study was carried out at Inamed Research GmbH and Co. KG, Gauting, Germany.

## Results

**Figure 2.**
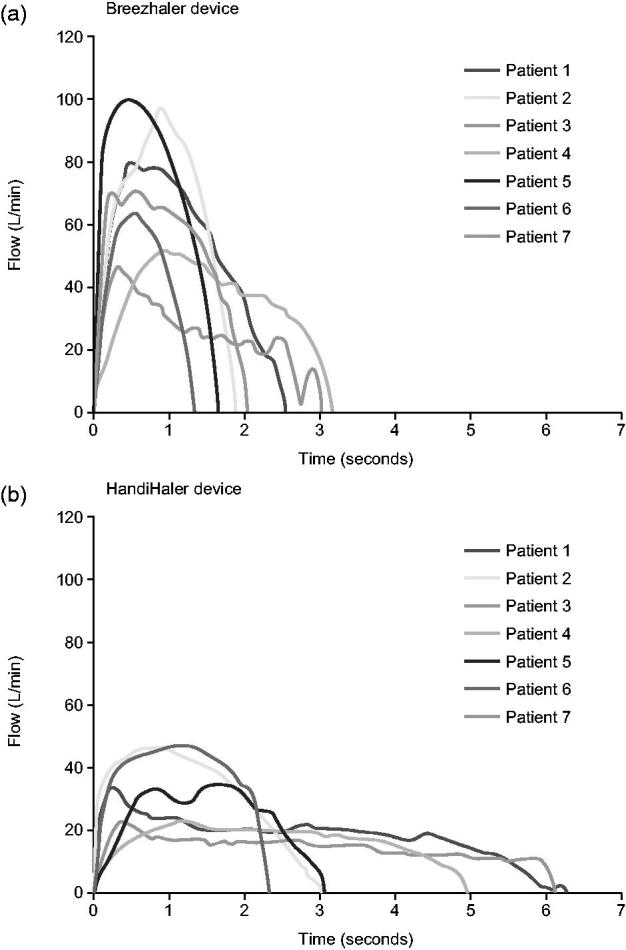
Individual inhalation flow profiles for the selected patients through the glycopyrronium capsule-based DPI (a) and tiotropium capsule-based DPI (b). Reproduced with permission from Chapman *et al*.^16^.

Seven breathing patterns derived from patients with moderate and severe COPD were reproduced to determine the dose delivery characteristics of glycopyrronium and tiotropium (Table 1 and Figure 2). The mean inhalation time (IT) was 2.2 s with the glycopyrronium capsule-based DPI and 4.2 s for the tiotropium capsule-based DPI (Table 1) while mean peak inspiratory flow (PIF) was 72 L/min and 36 L/min for the glycopyrronium and tiotropium capsule-based DPIs, respectively (Table 1 and Figure 2).

**Table 1.** Patient demographics and derived inhalation variables through the two inhalers.

Patient No.	Age	Gender	FEV_1_ (% pred.)	COPD	DP (cmH_2_O)		PIF (L/min)		IV (L)		IT (s)	
					GD	TD	GD	TD	GD	TD	GD	TD
1	74	Male	69	Mod.	24	31	80	34	2.2	1.9	2.5	6.3
2	69	Male	39	Sev.	35	58	97	47	2.1	1.6	1.9	3.0
3	79	Male	58	Mod.	8	14	47	23	1.3	1.5	3.0	6.1
4	70	Female	74	Mod.	9	15	48	24	1.7	1.4	3.2	5.0
5	52	Female	68	Mod.	37	44	99	41	2.0	1.8	1.7	3.8
6	76	Female	66	Mod.	15	34	64	36	1.0	1.3	1.3	3.1
7	71	Female	49	Sev.	19	61	72	48	1.8	1.5	2.0	2.3
Average	70		60		21	37	72	36	1.7	1.6	2.2	4.2

DP, pressure drop across the inhaler; FEV_1,_ forced expiratory volume in 1 second; GD, glycopyrronium capsule-based DPI; IT, inhalation time; IV, inhaled volume; PIF, peak inspiratory flow; TD, tiotropium capsule-based DPI. Data for the tiotropium capsule-based DPI were obtained from Chapman et al.^16^

The FPF ranged from 36.3% to 49.3% for glycopyrronium while it ranged between 7.6% and 10.9 % for tiotropium (Table 2). The mean FPF was 42.6% of the 50 µg nominal dose for glycopyrronium (RSD 10.68) and 9.8% of the 18 µg nominal dose for tiotropium (RSD 11.64) (Table 2). The MMAD was 2.8 µm for glycopyrronium (RSD 2.85) and 3.9 µm for tiotropium (RSD 6.33) (Table 2).

**Table 2.** Characteristics of aerosols generated using patient inhalation profiles representative of moderate to severe COPD.

Patient No.	Glycopyrronium capsule-based DPI					Tiotropium capsule-based DPI				
	DD	FPM	FPF	MMAD	GSD	DD	FPM	FPF	MMAD	GSD
	µg	µg	%	µm		µg	µg	%	µm	
1	41.6	22.6	45.2	2.8	1.9	7.9	1.7	9.6	3.9	1.9
2	41.8	22.4	44.9	2.7	1.9	8.0	1.9	10.4	3.7	1.8
3	38.4	18.2	36.3	2.8	2.0	6.7	1.4	7.6	4.4	1.8
4	39.3	19.1	38.1	2.9	1.9	7.8	1.8	10.0	4.2	1.8
5	43.5	24.6	49.3	2.7	1.9	6.9	1.7	9.4	3.8	1.8
6	37.9	20.2	40.4	2.7	1.9	8.2	2.0	10.9	3.8	1.8
7	40.4	22.1	44.2	2.7	1.9	7.8	2.0	10.9	3.9	1.9
Mean (SD)	40.4 (2.02)	21.3 (2.24)	42.6 (4.55)	2.8 (0.08)	1.9 (0.04)	7.6 (0.57)	1.8 (0.21)	9.8 (1.14)	3.9 (0.25)	1.8 (0.05)
RSD%	5.00	10.50	10.68	2.85	1.97	7.56	11.84	11.64	6.33	2.67

Data are means for each patient profile. DD, delivered dose (μg per capsule); FPM, fine particle mass (particles ≤4.7 μm in diameter); FPF, fine particle fraction (particles ≤4.7 μm in diameter) as % of nominal dose; GSD, geometric standard deviation; MMAD, median mass aerodynamic diameter; SD, standard deviation; RSD, relative standard deviation. Data for the tiotropium capsule-based DPI were obtained from Chapman *et al*.^16^.

**Figure 3.**
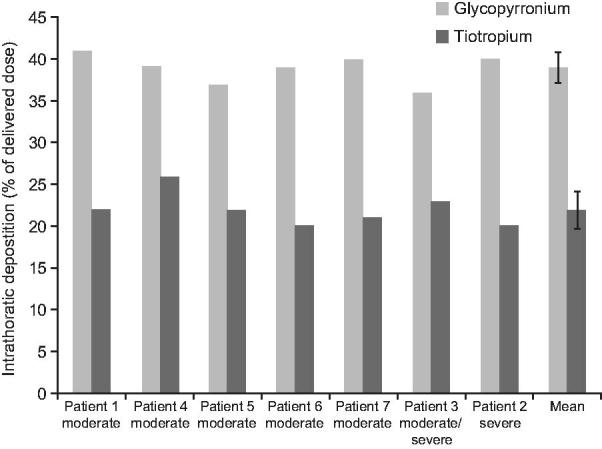
Theoretical intrathoracic drug deposition as percentage of delivered dose. Data for tiotropium were obtained from Chapman *et al*. 2011.

The mean estimated intrathoracic drug deposition as a percentage of the mean delivered dose was 39% (RSD 4.20) versus 22% (RSD 8.78) for glycopyrronium and tiotropium, respectively (Table 3 and Figure 3). The mean estimated extrathoracic drug deposition was 45% (RSD 3.02) for glycopyrronium and 71% (RSD 2.67) for tiotropium (Table 3).

**Table 3.** Theoretical intrathoracic and extrathoracic drug deposition as a percentage of delivered dose.

Patient No.	Glycopyrronium capsule-based DPI		Tiotropium capsule-based DPI	
	Extrathoracic deposition	Intrathoracic deposition	Extrathoracic deposition	Intrathoracic deposition
1	44	41	72	22
2	46	39	73	20
3	47	37	71	23
4	47	39	67	26
5	44	40	72	22
6	44	36	73	20
7	44	40	71	21
Mean (SD)	45 (1.36)	39 (1.64)	71 (1.90)	22 (1.93)
RSD %	3.02	4.20	2.67	8.78

Data are means for each patient profile. RSD, relative standard deviation; SD, standard deviation. Data for the tiotropium capsule-based DPI were obtained from Chapman *et al*.^16^.

## Discussion

In this study the impact of inhalation profiles representative of a range of COPD severity on the dose delivery characteristics of the LAMA glycopyrronium was evaluated and compared with results previously reported for tiotropium. Both glycopyrronium and tiotropium capsule-based DPIs delivered consistent doses across these inhalation profiles in terms of DD and FPF. However, delivery efficiency across COPD severity and ages for glycopyrronium (FPF from 36.3 to 49.3%) was higher than that for tiotropium (FPF from 7.6 to 10.9%). This finding is particularly important in light of the results of recent studies suggesting that older patients and patients with moderate-to-severe COPD have difficulty generating the necessary inspiratory flow to achieve an efficient and reproducible drug delivery from DPIs^8,9^. The internal resistance determines the inspiratory effort required to obtain consistent and efficient dose delivery from the inhalers^6,7^. Low-resistance devices allow air to flow through them easily as opposed to high-resistance devices. Therefore, they do not require a forceful and prolonged inhalation to achieve optimal drug delivery. As inspiratory flow is inversely proportional to inhaler resistance, the glycopyrronium capsule-based DPI (specific airflow resistances of 2.2 10^−2^ kPa_½_ L^−1^ min), may be more suitable for the majority of patients with COPD than the tiotropium capsule-based DPI (5.1 × 10^−2^ kPa_½_ L^−1^ min)^16^. Moreover, it has also been suggested that low-resistance DPIs have a greater patient acceptability^18^.

The predicted intrathoracic deposition of glycopyrronium (39%, RSD 4.20) was higher and more consistent than that previously estimated for tiotropium (22%, RSD 8.78) while the extrathoracic deposition was lower. In addition, the MMAD of the particles for glycopyrronium was smaller than that for tiotropium (2.8 µm and 3.9 µm, respectively). The higher FPF and intrathoracic deposition of glycopyrronium are not only determined by the device used to deliver this drug, but are also dependent on the formulation, which plays an important role in ensuring efficient delivery to the lungs^19^. The glycopyrronium formulation contains the excipient magnesium stearate as a ‘Force Control Agent’ which increases the dispersibility of drug particles thereby enabling optimal aerosolization^20^.

Particle size affects lung deposition, with a particle size of less than 4.7 µm in diameter generally considered optimal for deposition in the bronchi and alveoli^11^, The aerodynamic particle size distribution and the theoretical intra and extrathoracic deposition obtained for glycopyrronium in this study suggest that, compared with tiotropium, a higher proportion of the dose of glycopyrronium is delivered to the lower airways whereas a lower fraction deposits in the mouth and could be swallowed resulting in systemic exposure. This could in principle affect the clinical effectiveness of glycopyrronium as well as its tolerability profile. Nevertheless, it must be noted that FPF and the size of the particles generated by the inhalers are not a direct measure of lung deposition as they only provide an estimate of the dose likely to reach the lower airways. Moreover, the bronchodilator effect of inhaled drugs does not depend solely upon the percentage of the total dose that reaches the lung, but is the result of the combination of several physiological factors including airway geometry and lung clearance mechanisms^21^. Despite these limitations in extrapolating the current results to the clinical situation, a correlation between *in vitro* and *in vivo* data has previously been reported. A review of studies comparing the aerodynamic particle size measured *in vitro* at a constant air flow rate with lung deposition data obtained by gamma scintigraphy revealed that the aerodynamic particle size can predict the real distribution of inhaled drugs in the lung with reasonable accuracy^22^. Further, agreement between the delivered dose estimated in an *in vivo* study, which investigated the performance of pMDIs by simulating patient breathing profiles, and *ex vivo* data has previously been shown^23^. Thus the use of real inhalation profiles derived from patients with COPD in our study make the results obtained by this methodology even more predictive of the real drug delivery profiles in patients with COPD.

## Conclusions

This study provides new evidence to suggest that deposition in the lung is not always greater with high-resistance devices, as suggested for some DPIs^24,25^. The low-resistance glycopyrronium capsule-based DPI delivers a higher FPF and generates a greater and more consistent intrathoracic deposition compared with the tiotropium capsule-based DPI. Therefore, it may be suitable for patients with COPD of different severities, including severe COPD.

## Transparency

### Declaration of funding

This study was funded by Novartis Pharma AG, Basel, Switzerland.

### Declaration of financial/other relationships

P.C., T.K., E.C. and J.J. are employees of Novartis and declare no competing interests. T.V. has received reimbursement for attending scientific conferences and/or fees for presentations and/or consultations and/or educational programs from Boehringer Ingelheim, Chiesi, Janssen-Cilag, GlaxoSmithKline, Novartis, Teva and Mundipharma.

## Acknowledgments

The authors were assisted in the preparation of the manuscript by Roberta Sottocornola, a professional medical writer contracted to CircleScience (Macclesfield, UK) and Mark J. Fedele (Novartis). Writing support was funded by the study sponsor Novartis. The authors thank Inamed GmbH and Co. KG, Gauting, Germany who carried out the *in vitro* dose delivery study and analyzed the results. The authors also thank Dilraj Singh and Richard Pavkov from Novartis for the generation of the patient inhalation flow profiles.
